# Could Expanding and Investing in First-Episode Psychosis Services Prevent Aggressive Behaviour and Violent Crime?

**DOI:** 10.3389/fpsyt.2022.821760

**Published:** 2022-02-15

**Authors:** Sheilagh Hodgins

**Affiliations:** ^1^Département de Psychiatrie et Addictologie, Université de Montréal et Centre de Recherche de l'Institut Universitaire en Santé Mentale de Montréal, Montreal, QC, Canada; ^2^Haina Institute of Forensic Psychiatry, Haina, Germany

**Keywords:** first episode psychosis, treatment, prevention, aggressive behaviour, antisocial behaviour

## Abstract

**Objective:**

Some persons developing, or presenting, schizophrenia engage in aggressive behaviour (AB) and/or criminal offending. Most of these individuals display AB prior to a first episode of psychosis (FEP). In fact, approximately one-third of FEP patients have a history of AB, some additionally display other antisocial behaviours (A+AB). The large majority of these individuals have presented conduct problems since childhood, benefit from clozapine, have extensive treatment needs, and are unlikely to comply with treatment. A smaller sub-group begin to engage in AB as illness onsets. A+AB persists, often for many years in spite of treatment-as-usual, until a victim is seriously harmed. This article proposes providing multi-component treatment programs at FEP in order to prevent aggressive and antisocial behaviours of persons with schizophrenia.

**Method:**

Non-systematic reviews of epidemiological studies of AB among persons with schizophrenia, of the defining characteristics of sub-types of persons with schizophrenia who engage in AB and their responses to treatment, and of FEP service outcomes.

**Results:**

Studies have shown that mental health services that simultaneously target schizophrenia and aggressive behaviour are most effective both in reducing psychotic symptoms and aggressive behaviour. Evidence, although not abundant, suggests that a multi-component treatment program that would include the components recommended to treat schizophrenia and cognitive-behavioural interventions to reduce A+AB, and the other factors promoting A+AB such as substance misuse, victimisation, and poor recognition of emotions in the faces of others has the potential to effectively treat schizophrenia and reduce A+AB. Patients with a recent onset of AB would require few components of treatment, while those with prior conduct disorder would require all. Such a program of treatment would be long and intense.

**Conclusions:**

Trials are needed to test the effectiveness of multi-component treatment programs targeting schizophrenia and A+AB at FEP. Studies are also necessary to determine whether providing such programs in hospitals and/or prisons, with long-term community after-care, and in some cases with court orders to participate in treatment, would enhance effectiveness. Whether investing at FEP would be cost-effective requires investigation.

## Introduction

First-episode psychosis (FEP) services have improved clinical care by intervening early in the course of illness (1–3). By FEP, a significant minority of patients already have a history of aggressive behaviour (AB) and/or criminality. Among them, the largest group present a history of AB and antisocial behaviour (A+AB) since childhood, have the greatest treatment needs, and are the least likely to comply with treatment. A smaller group display AB that emerged as the prodrome progressed. Presently, such patients are not identified nor provided with the array of treatments required to reduce these behaviours as well as the symptoms of psychosis. Since many FEP services fail to assess and to treat A+AB, patients continue to assault others while in-and-out of psychiatric services. Finally, usually after several years, when a family member or carer, rarely a stranger, is severely injured, the perpetrator is charged with a violent crime. Some are judged to be not criminally responsible due to a mental disorder (NCRMD) and sent to a forensic hospital and others are found guilty and sentenced to incarceration in a correctional facility. The human and financial costs of this failure to identify and treat such patients when they first present to clinical services are huge. As this articles attempts to demonstrate, the extant scientific literature indicates that FEP services have the potential to prevent much A+AB and violent offending by persons developing or presenting schizophrenia. This would reduce human suffering of patients and victims and costs for police, courts, correctional facilities, social and health care. Further, it would reduce stigma against the mentally ill.

## Schizophrenia Confers an Increased Risk for Aggressive Behaviour

Much evidence confirms that individuals who are developing, or who present, schizophrenia are more likely than age and sex matched peers to engage in AB (4, 5). Some incidents of AB lead to criminal prosecution. Robust evidence collected in many different countries shows that people with schizophrenia are at increased risk, as compared to the general population where they live to be convicted of non-violent crimes, at higher risk to be convicted of violent crimes, and at even higher risk to be convicted of homicide [for a review see (6)]. Yet, mental health services for people with schizophrenia in most Western countries do not assess nor treat AB or the accompanying antisocial behaviours. While there are differences across jurisdictions, typically large proportions of patients in forensic psychiatric hospitals have long histories of treatment within general psychiatric services [see examples from the UK (7), Canada (8), The Netherlands (9), and across Europe (10)].

## Aggressive and Antisocial Behaviours and Crime Prior to Fep

A meta-analysis showed that 35% of individuals who contacted services for a first episode of psychosis had previously committed at least one assault (11), and subsequent studies of first-episode samples show similarly elevated rates of past AB (12, 13). For example, a study of 168 men and 133 women treated for a FEP in the UK found that one-third of the men and 10% of the women had been convicted or judged not guilty by reason of insanity of at least one offence, and 19.9% of the men and 4.6% of the women of at least one violent crime (14). A meta-analysis showed that the risk of homicide was 15.5 times higher among individuals experiencing a FEP who were not treated as compared to the general population (15).

While specialized services for first episode psychosis effectively treat schizophrenia, typically they do not assess nor treat AB or antisocial behaviour (1–3). Yet, by contact with mental health services for a FEP, most of the patients with schizophrenia who will offend have already begun to commit crimes (16), particularly crimes of violence, and/or to assault others. Importantly, these are the patients who will go on to commit the most A+AB and offences and to be the least compliant with treatment. This can be seen in a study of offending in the OPUS trial. Official criminal records of 547 FEP patients randomized to either assertive care (including family involvement, social skills training, staff/patient ratio 1/10) or standard outpatient care (staff/patient ratio 1/25) were compared (17). All patients received antipsychotic medication. Consistent with previous studies (11–14), prior to treatment, 32% of patients in the assertive program and 33% of patients in the traditional program had been convicted of crimes, 8% in each group of violent crimes. By the end of the five-year follow-up, 19% of the assertive program patients had acquired criminal convictions, 5% for violence, and 20% of the traditional program patients had acquired criminal convictions and 6% for violence. There was also no difference in the number offences committed by the two groups of patients. Also, consistent with previous evidence, three-quarters of the patients who acquired convictions during the five-year follow-up period had been convicted prior to treatment in the first-episode service. A US study compared criminality of 60 patients enrolled in a FEP program and 57 receiving treatment-as-usual. Like the OPUS program, the STEP program led to positive changes in schizophrenia symptoms. Unlike the OPUS trial, it may also have led to reductions in criminality (18). However, only convictions within the state of Connecticut at age 18 or older were examined, on average for 4 years before study entry and for six and one-half years after. Only 12% of patients had a criminal record prior to study entry, much lower than the proportions reported in other studies of FEP samples (11–14, 17). At the five-year follow-up, patients who received FEP treatment were non-significantly less likely than patients receiving treatment-as-usual to have acquired a conviction.

Overall, studies and meta-analyses show that approximately one-third of FEP patients have a history of A+AB and/or crime and thereby a very increased risk to continue to engage in A+AB and crime. Treatment focused on schizophrenia, even when the intensity is increased as in the OPUS trial (5, 17, 19), fails to reduce A+AB and crime.

## Characteristics of Patients Who Offend or Engage in AB Prior to a FEP

Among persons developing schizophrenia who offend or engage in AB prior to a first episode of psychosis are two distinct sub-groups. The largest sub-group, ~20–40% of persons with schizophrenia, present a history of conduct disorder that emerged in childhood (6). These individuals are responsible for most of the crimes committed by persons with schizophrenia. The severity of childhood conduct disorder symptoms is positively, and linearly, associated with the number of convictions for violent and non-violent crimes, and AB, even after taking account of substance misuse (20, 21). By adolescence these individuals are misusing substances (22), continuing to engage in AB, and displaying antisocial behaviour, attitudes, and ways of thinking.

Once psychosis onsets, patients with schizophrenia and prior conduct disorder show similar patterns of positive symptoms as other patients, but fewer negative symptoms (23–25). Among those with prior conduct disorder, positive symptoms are less related to AB than they are in other patients (23, 26). Many patients exhibit AB during an acute episode of psychosis that subsides as antipsychotic medication takes effect. In the Clinical Antipsychotic Trials of Intervention Effectiveness (CATIE) trial, antipsychotic medication was associated with a substantial reduction in AB but only among those without a childhood history of conduct problems (23). The CATIE trial did not test clozapine. Importantly, a recent randomized controlled trial demonstrated that patients with schizophrenia and prior conduct disorder showed greater reductions in psychotic symptoms and AB when treated with clozapine, as compared to olanzapine, and even greater reductions in AB as compared to haloperidol (27). The specific effect of clozapine was not observed among other patients. Clozapine has more impact on serotonergic brain circuits than the other antipsychotics and dysfunction in brain serotonin is associated with AB (28). This evidence adds to other findings suggesting that individuals who present conduct disorder prior to schizophrenia represent a distinct sub-type of the disorder. They show differences in brain morphology typical of people with schizophrenia and also differences typical of men without schizophrenia who present conduct disorder/antisocial personality disorder (29) and differences in social cognition (30). While few studies have been completed, the extant literature does suggest that FEP patients with a history of conduct disorder since childhood are distinct as to behaviour, negative symptoms, response to antipsychotic medication, and neural functioning.

A much smaller sub-group of persons developing schizophrenia present no history of conduct problems but begin engaging in AB as the prodrome onsets. Very little is known about these persons. In a study of men with schizophrenia recruited in general and forensic hospitals, those with and without a childhood conduct disorder included similar proportions who had been convicted of at least one violent crime, but, on average, those without prior conduct disorder had acquired fewer convictions for violent crimes and many fewer convictions for non-violent crimes. Importantly, however, a greater proportion of patients with no history of conduct disorder prior to illness onset, had been convicted for a homicide (23.9%) as compared to patients with prior conduct disorder (10.4%) [*X*2(N = 186) = 3.99, *p* = 0.046] (6). As would be expected, patients with no history of A+AB prior to illness onset were significantly older at first conviction for a violent crime than those with prior conduct disorder. Although it has been suggested that among those with no history of conduct disorder, substance misuse during the prodrome may promote AB (31, 32), in the study described above (6) there was no difference in the prevalence of substance use disorders among patients with and without conduct disorder.

Patient self-reports using standardized, validated questionnaires, and reports by older siblings and parents identify prior conduct problems and AB (20, 33). Official records can be obtained to document juvenile and adult offending. Such information distinguishes patients who began engaging in AB as the prodrome onsets from the larger sub-group who have presented conduct problems since childhood.

Thus, among patients presenting a FEP, two sub-groups can be identified, one with a long history of conduct problems, AB, sometimes criminal offending, and another with recent AB and sometimes violent, criminal offending. Patients in both sub-groups typically present comorbid substance use disorders.

## Aggressive and Antisocial Behaviours and Crime After FEP

A+AB and offending persist after FEP causing further suffering to perpetrators and victims and impacting general and forensic psychiatric services and the criminal justice system. Some of those who are prosecuted are judged not criminally responsible due to a mental disorder and held within forensic psychiatric hospitals. A recent study of all persons found not criminally responsible due to a mental disorder in British Columbia, Ontario, and Quebec Canada from 2000 to 2005 found that most were men (85%) with a diagnosis of schizophrenia (71%) prosecuted for a violent crime (8). Notably, three quarters of those with schizophrenia had been previously hospitalized in psychiatric services, on average five times, and just less than half had previously been found not criminally responsible due to a mental disorder or convicted of a crime (unpublished). The same finding emerged in a 1990 study (34). The persistent A+AB and offending after FEP is not only a problem in Canada but also elsewhere. For example, one study of men with schizophrenia recruited in general and forensic psychiatric services in Finland, Germany, Sweden, and Canada reported that 78% had previously been treated in general psychiatry (7). While some had committed their first crime before FEP, a smaller group committed their first offence after FEP but before the crime that led to admission to a forensic hospital. Most of the patients who committed crimes presented a history of childhood conduct disorder (7).

In some countries a small number of persons with schizophrenia are prosecuted and sentenced to a correctional facility (35) (see a UK example). In Canada, some of these persons receive a sentence of 2 years or longer of incarceration in a penitentiary. Diagnostic studies of random samples of male penitentiary inmates reported that in 1995 6.3% presented schizophrenia (36) and in 2016 4.7% presented psychotic disorders (37), while the prevalence among female inmates varied from 9.4 to 5.8% (38).

General psychiatric services treat many patients with schizophrenia who present A+AB. For example, a study of 205 patients with psychosis hospitalized in a general psychiatry service in a UK university hospital, found that 63.3% of the men and 23.5% of the women had been convicted for non-violent crimes, and 46.7% of the men and 16.5% of the women for violent crimes. The male offenders had been convicted, on average, for 10.7 non-violent crimes and for two violent crimes. The female offenders had been convicted, on average for 9.7 non-violent crimes, and two violent crimes. The male patients were three times more likely than men in the general UK population to be convicted of non-violent crimes and five times more likely to be convicted of violent crimes. The female patients were three times more likely than women in the UK to be convicted of non-violent crimes and 17 times for violent crimes. In similar comparisons with Swedish and Danish patient and general population samples, elevations in risk of offending among male patients were similar, while the risk of violence was higher among the female patients in the UK. In this UK sample of inpatients, 52% of the men and 31% of the women self-reported AB, and 21% of the men and 15% of the women reported AB that seriously injured another (4). Patients with a history of childhood conduct disorder were the most likely to have committed AB and violent crimes (20). Two years later, clinical files and patient reports indicated that the patients with criminal records had not received any treatments aimed at reducing criminality or AB, nor treatments aimed at reducing substance misuse (19). However, clinical files indicated that the frequency of meetings with the care team was significantly and positively associated with the number of incidents of AB during the past 6 months and with illicit drug use. Again, as in the OPUS trial and other studies (5, 17), increased intensity of clinical contact while not providing treatments that target AB+B and promote prosocial behaviour failed to prevent criminal offending.

## Additional Factors Promoting AB

People developing, and presenting, schizophrenia are more likely than their healthy peers to misuse substances (6). For example in a Swedish study, 24% of the 4674 persons with schizophrenia misused substances as compared to 5% of their healthy siblings, and among those with schizophrenia, substance use disorders were associated with a 1.8 fold increase in risk of violent crime relative to siblings, and a 4.4 fold increase relative to healthy age and sex matched peers (39). A meta-analysis showed that in samples of patients treated in FEP clinics, between 29 and 38% used cannabis (40). Prior to legalization of cannabis in Canada, a study of a sample of patients with recent diagnoses of psychosis reported that 13% were misusing alcohol-only, 21% cannabis-only, and 50% both alcohol and cannabis (41). Cannabis may be specifically linked to AB among persons with severe mental illness. A recent meta-analysis found that among persons with severe mental illness the risk of AB was increased two-fold by cannabis use and five-fold by the presence of cannabis use disorder (42). However, most studies showing a link between substance misuse and AB have failed to take account of prior conduct disorder or AB. Other studies show that there is no association of substance misuse with AB after controlling for childhood conduct problems (20, 21, 23, 31, 43). Among patients with prior conduct disorder, substance misuse onsets early in adolescence (22) and becomes an integral part of an antisocial life-style. Among a smaller group, substance use may be an attempt to reduce the increasing symptoms prior to FEP.

From childhood onwards, persons with schizophrenia have problems recognizing emotions in the faces of others from childhood onwards (44, 45). Poor recognition especially of fear and anger (46) has been associated with AB (47, 48).

Another factor linked to AB among persons with schizophrenia is physical victimisation. Many studies show that children/adolescents developing schizophrenia are significantly more likely to experience physical and/or sexual maltreatment, and bullying than their healthy peers (49). Among patients with schizophrenia, those presenting prior conduct disorder are the most likely to have experienced maltreatment (33). Adults with schizophrenia, both those with and without prior experiences of victimization, show higher levels of victimization than their neighbours (50), even after taking account of their own criminality (51), and an even higher risk of being a victim of homicide (52). A study of a large cohort of Swedes demonstrated that among people with schizophrenia, violent victimization was associated with AB in the following week (53).

## What is Known About Treatments to Reduce A+Ab and Offending Among Persons with Schizophrenia?

In-hospital (54) or in-prison (55) multi-component treatment programs reduce both psychotic symptoms and A+AB. For example, one study followed matched samples of men with schizophrenia discharged from general and forensic psychiatric hospitals. After 2 years, the forensic patients, as compared to the general psychiatry patients, displayed lower levels of positive and negative symptoms and higher levels of psychosocial functioning. During the two-year follow-up, similar proportions of general and forensic patients were re-hospitalized, and significantly fewer of the forensic (12%) than the general patients (29%) engaged in AB (7, 56). A similar study in Quebec again showed that treatment by a forensic service achieved more effective reductions in psychotic symptoms and AB than that provided by general psychiatric services (57). Thus, clinical services that focused on both the schizophrenia and the A+AB were more effective in treating schizophrenia and preventing AB than those that focused only on schizophrenia. Although there is no direct evidence, it could be that good outcomes were due not only to the intensity and array of interventions but also to the length of treatment, the involuntary hospitalization/imprisonment, and that discharge depended on a patient's demonstrating a low risk of future A+AB.

Given the knowledge about the timing of AB and offending among persons with schizophrenia, FEP clinics have the potential to intervene and prevent much, if not all, subsequent A+AB and offending. This would require focusing treatment on schizophrenia as well as on A+AB. Consider such a multi-component treatment program (see Table 1). The results of randomized controlled trials show that the effective treatment of schizophrenia includes antipsychotic medication, psychoeducation, and life-skills and social-skills training (1). Additionally, trials have shown that patients benefit from cognitive rehabilitation programs (58–60) and supportive employment programs (61). For patients misusing substances, treatment programs integrated into their care programs have been shown to be effective (62) and they could be tailored to individual patient needs. Since victimization has been shown to trigger AB (53), monitoring victimization and teaching patients to avoid situations where such victimisation occurs would be beneficial. Further, programs that improve patients' abilities to recognize emotions in the faces of others have been associated with reductions in AB (48), and may also facilitate social interactions. Additional components of treatment would be required for patients presenting a history of A+AB and/or offending, especially those with prior conduct disorder, including cognitive-behavioural programs designed to reduce AB and antisocial behaviour, attitudes, and ways of thinking (63). Clinical trials are needed to measure the effectiveness of such multi-component treatment programs.

**Table 1:** Assessment and treatment of schizophrenia, aggressive and antisocial behaviour at first-episode of psychosis.

	**History of aggressive behaviour**		**No history of aggressive behaviour**
	**Aggressive behaviour since childhood**	**Aggressive behaviour since prodrome**	
	**Assessment**		
Symptoms (onset, severity, etc)	✓	✓	✓
Interpersonal functioning	✓	✓	✓
Recognizing emotions in faces of others	✓	✓	✓
Life skills	✓	✓	✓
Social skills	✓	✓	✓
Employment skills	✓	✓	✓
Cognitive performance	✓	✓	✓
Aggressive behaviour (onset, frequency, severity)	✓	✓	✓
Antisocial behaviour (onset, severity)	✓	✓	✓
Individual risk factors for aggressive behaviour	✓	✓	
Individual protective factors against aggressive behaviour	✓	✓	
Substance misuse (onset, types, frequency)	✓	✓	✓
Compliance with antipsychotic medication	✓	✓	✓
Estimated compliance with behavioural and/or cognitive behavioural programs	✓	✓	
	**Treatment**		
Antipsychotic medication	✓ Clozapine	✓	✓
Psychoeducation about schizophrenia	✓	✓	✓
Life-skills training	As needed	As needed	As needed
Social skills training	As needed	As needed	As needed
Cognitive rehabilitation	As needed	As needed	As needed
Training to recognize emotions in faces of others	As needed	As needed	As needed
Employment training or supportive employment	✓	✓	✓
Substance misuse treatment adapted to patient profile	✓	✓	If needed
Cognitive-behavioural treatment for aggressive behaviour	✓	✓	
Cognitive-behavioural treatment for antisocial behaviour, attitudes, and ways of thinking	✓		
Learning to avoid and report victimisation	✓	✓	✓
Court order to ensure compliance with multiple treatment components	✓ Probably needed		
Regular assessment of risk and protective factors	✓	✓	

The accumulated evidence suggests that multi-component treatment programs that are tailored to individual patient needs would last many months followed by community care that also focused on both schizophrenia and A+AB. Patients with prior conduct disorder might benefit if the program was initially offered in a hospital, or within a special unit in a correctional facility for those who have committed crimes.

Encouraging patients with a history of A+AB to engage with lengthy, intensive treatment programs is a challenge. We tried and failed (64)! We conducted a feasibility study that attempted to use the Reasoning & Rehabilitation Programme (R&RP) to reduce aggressive behaviour among men with severe mental illness who were receiving out-patient mental health care and living independently in the community. The R&RP includes 38 two-hour sessions offered three times a week, and prior to our study it had been shown to reduce criminality among non-mentally ill offenders (65).

A research worker interviewed each of the 28 participants before treatment began, every month for the subsequent 6 months, and after the planned termination of treatment. Patients were paid £5.50 for each interview. Patients were randomly assigned to R&RP plus their usual community care or only their usual community care. Therapists contacted participants before the first R&RP session and after each missed session to encourage participation and to provide practical information. As we observed the lack of participation by patients in the RR&P, we offered two additional sessions of motivation therapy to the last 10 patients. The average time spent by therapists engaging each patient in the R&RP was 2.9 h (SD = 2.6) and the total time required for training, preparation, reminders, motivational interviews and delivery of R&RP was 372.6 h.

Of the 28 patients, 25 attended no R&RP session and three attended one session. Five patients had been readmitted to hospital and were not given leave from the ward to attend, nine were too busy, four were confused or afraid of attending, and no reasons for failure to attend were provided by the others. By contrast, all participants completed a lengthy interview at study entry, and one-third to one-half completed monthly interviews for which they were paid £5.50 for each interview. During the 6 month follow-up period, according to file and/or self-reports, four (14.3%) patients were convicted of criminal offences, 16 (57.1%) were in contact with the police, 10 (35.7%) engaged in at least one physically aggressive behaviour towards another person, and nine (32.1%) experienced physical victimisation. Four (14.3%) patients reported misusing alcohol and 12 (42.9%) reported using illicit drugs. All were partially or fully compliant with meetings with their community treatment team, with an average of 12 (SD = 7.0) meetings during the follow-up period. In conclusion, patients with severe mental illness failed to attend R&RP, many completed research interviews for which they were paid, and all attended regular meetings with care co-ordinators who assured that their social benefits continued.

A randomized, controlled, trial focused on the R&RP among men with psychotic disorders in a forensic hospital. Only half of the patients allocated to receive R&RP completed the programme. *Post-hoc* analyses of completers vs. non-completers determined that programme completers showed improvements in social problem-solving at the end of treatment and changes in criminal attitudes at 12 months post-treatment, and after controlling for psychopathic traits, fewer incidents of violence, verbal aggression, and leave violations during treatment, and less verbal aggression and substance use at 12-months posttreatment (66, 67). A modification of the R&RP for persons with mental disorders has been tested in a small sample of forensic inpatients and has obtained higher participation rates than previous studies (63). These results confirm that the challenge is to identify strategies for encouraging participation in lengthy, intensive, treatment programs (68).

Trials are also needed to identify effective strategies for ensuring participation and completion of lengthy and intense treatment programs. Studies of forensic assertive case management, mental health courts, outpatient commitment orders, jail diversion and re-entry programs either showed a small positive effect or no effect on compliance with antipsychotic medication and A+AB and/or criminality. Most studies, however, did not take account of the treatment provided that was generally very limited [for reviews see (69–73)]. Mental health courts and outpatient commitment orders could be used to encourage participation and completion of such a program of treatment.

## Conclusion

All the available evidence suggests that identifying and treating A+AB at FEP, in addition to psychosis, would reduce suffering and costs and promote independent, safe, community tenure for patients. In addition to trials to test these propositions, cost-benefit studies of intervening at FEP are needed. Funds currently spent on mental and physical care of victims, policing, courts, forensic hospitals, review boards, prisons and penitentiaries could be used to establish effective treatment programs at FEP. Suffering, stigma, and costs could also be reduced by further research and interventions aimed at early-intervention (74). In a meta-analysis of data from studies of clinical-high-risk patients, obsessive–compulsive symptoms and AB distinguished those who transitioned to psychosis (75). Early intervention for psychosis and prevention of AB and offending could also be achieved by screening juvenile delinquents and substance misusing youth for antecedents of schizophrenia. Among adolescents who commit offences or who are in treatment for substance misuse, ~4–7% subsequently develop schizophrenia (76, 77). The current practice of waiting until persons with schizophrenia who present long histories of psychotic illness and A+AB seriously harm another is a waste of human and financial resources.

## Author Contributions

The author confirms being the sole contributor of this work and has approved it for publication.

## Funding

This work was supported by the Haina Institute of Forensic Psychiatry.

## Conflict of Interest

The author declares that the research was conducted in the absence of any commercial or financial relationships that could be construed as a potential conflict of interest.

## Publisher's Note

All claims expressed in this article are solely those of the authors and do not necessarily represent those of their affiliated organizations, or those of the publisher, the editors and the reviewers. Any product that may be evaluated in this article, or claim that may be made by its manufacturer, is not guaranteed or endorsed by the publisher.
